# Co-occurrence of autoimmune diseases and metabolic disorders in familial mediterranean fever patients: review of literature and case reports

**DOI:** 10.3389/fimmu.2025.1726664

**Published:** 2025-12-15

**Authors:** Ahlam Chaaban, Nohad Baalbaki, Ralph Narch, Eliana Eldawra, Philippe Hussein Kobeissy, José-Noel Ibrahim

**Affiliations:** Department of Biological Sciences, School of Arts and Sciences, Lebanese American University (LAU), Beirut, Lebanon

**Keywords:** familial mediterranean fever, fmf, autoinflammatory disease, autoimmune diseases, metabolic disorders, *MEFV*

## Abstract

Familial Mediterranean Fever (FMF) is an autosomal recessive autoinflammatory disease predominantly affecting populations from the Mediterranean region, with a high prevalence reported among Armenians, Turks, Arabs, and Jews. The condition results from mutations in the *MEFV* gene, which encodes pyrin, a key regulator of inflammation. Both genetic and epigenetic factors contribute to the diverse clinical manifestations of FMF, which are characterized by recurrent episodes of fever, abdominal pain, chest pain, arthritis, and erysipelas-like erythema. In addition to these symptoms, individuals with FMF patients appear to have an increased risk of developing autoimmune and metabolic disorders, likely due to shared inflammatory pathways. In fact, FMF exemplifies a monogenic autoinflammatory disorder arising from mutations in genes related to the innate immune system, unlike autoimmune diseases that stem from defects in the adaptive immune system. Despite differences in their underlying mechanisms, both autoinflammatory and autoimmune diseases involve the production of interleukin-1β, a key cytokine that influences effector cells of the adaptive immune system, including B and T lymphocytes. Accordingly, numerous studies have investigated the increased prevalence of autoimmune and metabolic disorders among FMF patients, seeking to understand whether these comorbidities exacerbate FMF symptoms, increase the risk of complications, or affect treatment responses. This review examines the coexistence of FMF with autoimmune diseases and metabolic disorders and explores potential correlations with *MEFV* mutations.

## 1 Autoinflammatory vs. autoimmune diseases

Autoinflammatory diseases (AIDs) are clinical disorders that arise from dysregulation of the innate immune system. These conditions are characterized by recurrent or persistent episodes of inflammation, with little to no involvement of the adaptive immune system in disease pathogenesis (1). In fact, AIDs often involve mutations in genes related to cytokines and inflammasomes, protein complexes that activate the innate immune system, and may present with recurrent episodes of fever among other systemic inflammatory symptoms (2). In contrast, autoimmune diseases (ADs) are primarily mediated by autoantibodies or autoreactive B and T cells, leading to chronic inflammation and localized tissue destruction (3). Despite their distinct pathophysiology, AIDs and ADs exhibit overlapping features in their genetic background, inflammatory manifestations, and clinical presentations (3). Both diseases can cause persistent inflammation throughout the body in the absence of an infectious trigger, leading to a range of symptoms such as pain, swelling, fever, and fatigue, ultimately resulting in a chronic condition that necessitates ongoing medical management (3). AIDs comprise a broad and continuously expanding spectrum of disorders (1). Within this spectrum, one category consists of polygenic AIDs, which include complex multifactorial disorders of unknown etiology, with no clearly identified genetic mutations (2). Another category, monogenic autoinflammatory diseases (mAIDs), consists of rare genetic immune disorders arising from mutations in genes linked to innate immune pathways. Among them, inflammasomopathies, or inflammasome-mediated AIDs, are caused by inherited defects in proteins that form inflammasome complexes.

**Figure 1:**
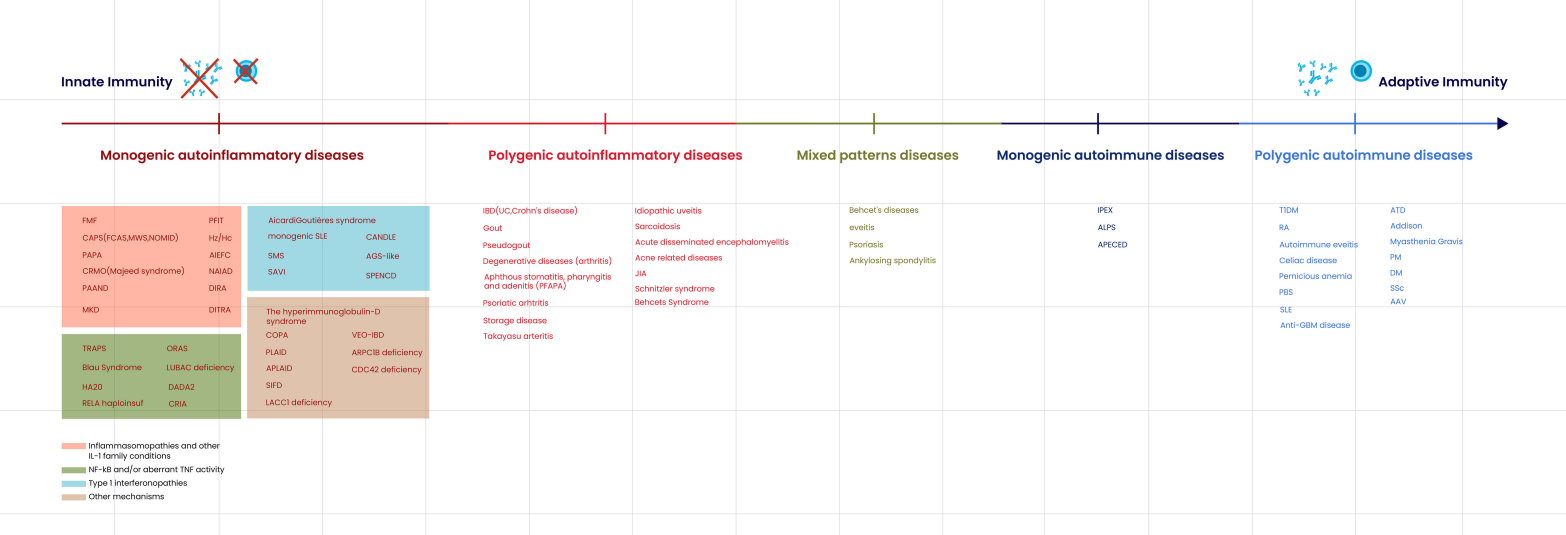
The spectrum of immune-mediated diseases based on the degree of genetic contribution—ranging from monogenic to polygenic—and the type of immune response involved, from innate to adaptive immunity. On the left, monogenic autoinflammatory diseases are primarily driven by innate immune dysregulation. Moving rightward, the spectrum includes polygenic autoinflammatory diseases and disorders with mixed immune mechanisms, reflecting increasing complexity in both genetic and immunological factors. Further along the continuum are monogenic autoimmune diseases, predominantly mediated by adaptive immune responses involving autoantibodies and/or autoreactive B and T cells, with polygenic autoimmune diseases positioned at the far right. Monogenic autoinflammatory diseases are categorized based on their underlying mechanisms, including inflammasomopathies and other IL-1 family-related conditions (red), NF-κB pathway abnormalities and/or excessive TNF activity (green), type I interferonopathies (blue), and other mechanisms (brown). FMF, Familial Mediterranean Fever; CAPS (FCAS, MWS, NOMID), Cryopyrin-Associated Periodic Syndromes (Familial Cold Autoinflammatory Syndrome, Muckle–Wells Syndrome, Neonatal-Onset Multisystem Inflammatory Disease); PAPA, Pyogenic Arthritis, Pyoderma Gangrenosum, and Acne syndrome; CRMO, Chronic Recurrent Multifocal Osteomyelitis (Majeed syndrome); PAAND, Pyrin-Associated Autoinflammation with Neutrophilic Dermatosis; MKD, Mevalonate Kinase Deficiency; TRAPS, TNF Receptor-Associated Periodic Syndrome; Blau syndrome, Granulomatous arthritis/uveitis/dermatitis due to NOD2 mutation; HA20, Haploinsufficiency of A20; RELA haploinsufficiency, NF-κB p65 haploinsufficiency; CRIA, Cleavage-Resistant RIPK1-Induced Autoinflammatory syndrome; PFIT, Periodic Fever, Immunodeficiency, and Thrombocytopenia; Hz/Hc, Hypozincemia/Hypercalprotectinemia; AIEFC, Autoinflammation with Episodic Fever and Colitis; NAIAD, NF-κB Activation Impairment with Dysregulated Cytokine production syndrome; DIRA, Deficiency of the Interleukin-1 Receptor Antagonist; ORAS, Otulipenia/ORAS (OTULIN-Related Autoinflammatory Syndrome); LUBAC deficiency, Linear Ubiquitin Chain Assembly Complex deficiency; DADA2, Deficiency of Adenosine Deaminase 2; DTRA, Deficiency of TNF Receptor Antagonist; Aicardi–Goutières syndrome (AGS), Type I interferonopathy; Monogenic SLE, Monogenic Systemic Lupus Erythematosus; CANDLE, Chronic Atypical Neutrophilic Dermatosis with Lipodystrophy and Elevated temperature; SMS, Singleton–Merten syndrome; SAVI, STING-Associated Vasculopathy with Onset in Infancy; SPENCD, Spondyloenchondrodysplasia with Immune Dysregulation; COPA, COPA syndrome (autoimmune interstitial lung disease/arthritis); VEO-IBD, Very Early Onset Inflammatory Bowel Disease; PLAID, PLCγ2-Associated Antibody Deficiency and Immune Dysregulation; APLAID, Autoinflammation and PLCγ2-Associated Antibody Deficiency; SIFD, Sideroblastic Anemia with Immunodeficiency, Fevers, and Developmental Delay; LACC1 deficiency, Laccase Domain-Containing 1 deficiency; ARPC1B deficiency, Actin-Related Protein 2/3 Complex Subunit 1B deficiency; CDC42 deficiency, Cell Division Control Protein 42 deficiency; IBD (UC, Crohn’s disease), Inflammatory Bowel Disease (Ulcerative Colitis, Crohn’s disease); JIA, Juvenile Idiopathic Arthritis; PFAPA, Periodic Fever, Aphthous Stomatitis, Pharyngitis, Adenitis syndrome; SLE, Systemic Lupus Erythematosus; SSc, Systemic Sclerosis; RA, Rheumatoid Arthritis; T1DM, Type 1 Diabetes Mellitus; PBS, Primary Biliary Sclerosis; IPEX, Immune Dysregulation, Polyendocrinopathy, Enteropathy, X-linked syndrome; ALPS, Autoimmune Lymphoproliferative Syndrome; APECED, Autoimmune Polyendocrinopathy–Candidiasis–Ectodermal Dystrophy; ATD, Autoimmune Thyroid Disease; PM, Polymyositis; DM, Dermatomyositis; AAV, ANCA-Associated Vasculitis; Anti-GBM disease, Anti–Glomerular Basement Membrane disease.

**Figure 1** illustrates the spectrum of immune-mediated diseases based on the degree of genetic contribution—ranging from monogenic to polygenic—and the type of immune response involved, from innate to adaptive immunity.

## 2 Familial mediterranean fever

### 2.1 History and prevalence

Familial Mediterranean Fever (FMF) is the most recognized and prevalent AID worldwide (4). It was first described in 1945 by allergist Siegal (5). Initially identified as ‘benign paroxysmal peritonitis,’ it was later referred to as periodic peritonitis, familial recurrent polyserositis, and periodic disease. The modern universally accepted name, ‘Familial Mediterranean Fever,’ was coined by Heller in 1958 (6). While populations from the Eastern Mediterranean, including Turks, Jews, Arabs, and Armenians, are particularly susceptible to FMF, numerous cases have been documented in Europe, North America, and Japan due to migration from endemic regions (7). The prevalence of FMF ranges from 1 in 500 to 1 in 1,000 in endemic regions, with Central Anatolia reporting the highest recorded prevalence of 1 in 395 (8).

### 2.2 Genetics and pathophysiology

FMF is an autosomal recessive disease caused by mutations in the *MEFV* (MEditerranean FeVer) gene, which is located on the short arm of chromosome 16 (16p13.3). The gene consists of 10 exons, with over 404 variants recorded to date (9). The majority of the variants classified as pathogenic or likely pathogenic are found in exon 10. The most prevalent variants in FMF-endemic regions are the M694V, M694I, V726A, and M680I, located in exon 10, along with the E148Q variant in exon 2, collectively accounting for 85% of cases (8, 10). Genotype-phenotype correlation studies indicate that M694V mutation is associated with the most severe phenotype of the disease while the clinical significance of E148Q — whether as a pathogenic variant with low penetrance or a benign polymorphism — remains under investigation (11).

The *MEFV* gene encodes a 781 amino acid protein called pyrin, also known as marenostrin, which is mainly expressed in granulocytes, eosinophils, monocytes, and dendritic cells. Pyrin consists of five domains: the N-terminal Pyrin domain (PYD), bZIP transcription factor domain, B-box zinc finger, α-helix coiled-coil domain, and the C-terminal B30.2/SPRY domain (12). *MEFV* mutations, predominantly clustered in the B30.2 domain, activate pyrin which associates with pro-caspase-1 and apoptosis-associated speck-like protein (ASC) to form a multiprotein complex known as inflammasome. The assembly of the pyrin inflammasome leads to the autocatalytic activation of caspase-1, which subsequently converts pro-interleukin-1beta (IL-1β) and pro-IL-18 into their active forms, IL-1β and IL-18. Caspase-1 also mediates pyroptosis through the gasdermin D pathway leading to the formation of membrane pores and the release of pro-inflammatory cytokines, thereby amplifying ongoing subclinical inflammation (12, 13).

### 2.3 Clinical manifestations

FMF typically manifests before the age of 20, with the majority of cases reported during childhood (4). A less common adult-onset presentation of FMF is reported in some patients, who tend to have milder clinical manifestations (14). FMF is characterized by recurrent bouts of fever and inflammation, frequently affecting the joints and serosal membranes. In fact, increased IL-1β production results in subclinical inflammation persisting between attacks in patients with FMF (15, 16). The frequency of episodes varies among individuals, occurring as often as once a week or as infrequently as every few months. Several factors were reported to trigger FMF attacks, including, stressful situations, exposure to cold, and hormonal changes in women during menstrual cycles (17). Nearly half of patients with FMF experience a prodrome, which precedes the attacks and is marked by discomfort, malaise, and occasional neurological symptoms such as headaches (17).

During an attack, fever typically lasts 12 to 72 hours and reaches temperatures of 38 °C to 40 °C. One of the most common symptoms of FMF is intense joint pain, with monoarthritis being the most frequent manifestation (4). Although arthritis usually resolves within 24 to 48 hours, it can occasionally cause severe joint damage (4). Abdominal pain is another common symptom, often severe enough to mimic an acute abdomen. It may be associated with diarrhea or constipation due to sterile peritoneal inflammation (18). Furthermore, pericardial and pleural inflammation may result in dyspnea, pleural effusion, and chest pain (19). Although rare, pericarditis occurs more frequently in patients with FMF than in the general population (4). Other reported FMF manifestations include erysipelas-like erythema, recurrent oral ulceration, myalgia, and orchitis (17, 20). Finally, the most serious complication of untreated or improperly managed FMF is amyloidosis, characterized by the buildup of amyloid proteins in organs, including the kidneys, resulting in renal failure (21). In this regard, monitoring and controlling inflammation are crucial to avoid long-term consequences (21). The clinical presentation of FMF varies significantly among patients. In addition to *MEFV* allelic heterogeneity, modifying genes such as *IL-1β* (22) and *SAA1* (Serum Amyloid A1) (23), as well as environmental factors, contribute to the variable expressivity of the disease through epigenetic mechanisms, primarily DNA methylation and microRNAs (miRNAs) (24). Nonetheless, few characteristic traits remain usually evident, aiding in diagnosis and treatment.

### 2.4 Treatment

Colchicine, extracted from the plant *Colchicum autumnale*, is the oldest known medication still used today to treat patients with FMF (25). It is effective in reducing the frequency and severity of attacks and in preventing the development of amyloidosis. Although its capacity to bind microtubules and prevent mitosis was first established, current studies support the existence of additional mechanisms of action of colchicine in FMF treatment. Indeed, studies have shown that colchicine impairs neutrophil chemotaxis, lowers leukocyte function, and attenuates inflammation through suppression of pyrin and NLRP3 inflammasomes (26). Colchicine is generally safe, but it has a limited therapeutic index and is associated with gastrointestinal symptoms such as cramps, diarrhea, and vomiting, all of which are common adverse effects (27). Notably, 5-10% of patients exhibit partial or no response to colchicine treatment (28). Classified as colchicine-resistant, this category of patients can benefit from alternative treatments including the administration of IL-1β inhibitors, namely, anakinra (an IL-1 receptor antagonist) and canakinumab (a human monoclonal anti-IL-1β antibody) (29).

## 3 Comorbidities in FMF

The presence of comorbidities in FMF may contribute to the heterogeneity of clinical manifestations, influence disease severity, and affect patients’ responses to treatment (30). This may explain why some individuals require specialized management and face therapeutic challenges. Supporting this, a large cross-sectional study of 686 pediatric FMF patients reported that around one-fifth of the patients had coexisting inflammatory conditions—such as juvenile idiopathic arthritis, asthma, Henoch–Schönlein purpura, uveitis, and inflammatory bowel disease— highlighting the frequent overlap between FMF and other immune-mediated disorders (31). Therefore, to improve the quality of life and long-term health outcomes for FMF patients, researchers aim to unravel the complex interactions between FMF and coexisting comorbidities, with the goal of developing more targeted therapeutic strategies.

Consequently, the objective of this review is to comprehensively examine the relationship between FMF and its co-occurring disorders—specifically, autoimmune and metabolic diseases — focusing on *MEFV* variants, underlying shared pathogenic mechanisms, and clinical presentation. To achieve this aim, a thorough literature search was conducted using databases such as PubMed, Google Scholar, Scopus, and Medline. Relevant studies published in English within the last 10–15 years were identified using the following keywords: “Familial Mediterranean Fever”, “FMF”, “*MEFV*”, “autoimmune diseases/disorders”, “metabolic diseases/disorders”, “Inflammatory Bowel Disease”, “IBD”, “Systemic Lupus Erythematosus”, “SLE”, “Multiple Sclerosis”, “MS”, “Rheumatoid Arthritis”, “RA”, “Sjögren’s Syndrome”, “SS”, “Celiac Disease”, “CD”, “Hashimoto’s Thyroiditis”, “HT”, “Type 1 Diabetes”, “T1D”, “Metabolic Syndrome”, “MetS”, “Non-Alcoholic Fatty Liver Disease”, and “NAFLD”. Studies assessed included original research articles, review articles, case reports, and letters to the editor. After initial screening of titles and abstracts, full texts were reviewed to confirm relevance. Data on study design, population characteristics, and key findings were extracted, synthesized, and interpreted, and existing gaps were identified to help guide future research directions.

### 3.1 FMF and autoimmune diseases

#### 3.1.1 FMF and inflammatory bowel disease

Inflammatory bowel disease (IBD) is a complex immune-mediated disease characterized by chronic inflammation, damage to the epithelium of the gastrointestinal tract, abnormalities in innate immune responses, and dysregulation of the adaptive immune system (32). Typically, IBD arises in early childhood and adulthood and manifests in three forms: Crohn’s disease (CD), ulcerative colitis (UC), and inflammatory bowel disease unclassified (IBDU), previously referred to as indeterminate colitis (IC) (32).

FMF and IBD share several clinical and pathogenic features, which have been extensively explored in the literature. First, both conditions are associated with inflammasome pathway dysregulation, though through different mechanisms. While FMF is associated with mutations in the *MEFV* gene and the activation of the pyrin inflammasome, IBD is a more complex disorder linked to mutations in *NLRP3*, which encodes the cryopyrin protein involved in inflammasome-mediated IL-1β processing (33). Interestingly, wild type pyrin was found to negatively modulate the NLRP3 inflammasome by interacting with several components of the inflammasome, mainly ASC, to inhibit pro-IL-1β processing (34, 35). Beyond its role in inflammasome regulation, pyrin also contributes to autophagy by facilitating the degradation of inflammasome components such as NLRP3 and pro-caspase 1. Dysfunction in this autophagic process has been strongly linked to the development of IBD (36, 37). Furthermore, the caspase activation recruitment domain family member 15 (*CARD15*) gene, which encodes the nucleotide-binding oligomerization domain-containing protein 2 (NOD2), plays a key role in cellular protection against invasive bacteria via the activation of the NF-kB (nuclear factor-kappa B) pathway. In CD, NOD2 acts as a susceptibility gene; abnormal responses to bacterial components can disrupt innate immune signaling, resulting in persistent intestinal inflammation (38, 39). Remarkably, pyrin also regulates neutrophil activity through its CARD domain, supporting the idea that pyrin and the *NOD2/CARD15* gene product belong to the same death domain superfamily involved in cytokine processing and inflammatory signaling (39). These genetic and molecular parallels have encouraged researchers to explore the connection between FMF and IBD, particularly the role of *MEFV* mutations in IBD pathogenesis.

A recent large-scale, population-based study from Israel using the epi-IIRN cohort and covering 98% of the national population showed a significantly higher prevalence of FMF among patients with IBD compared to matched non-IBD controls (40). Specifically, FMF was more common in CD patients (0.6%) than in those with UC (0.3%). Notably, in 83% of cases, FMF was diagnosed prior to IBD onset. The study also found that FMF was associated with increased disease severity at diagnosis in UC but not in CD. UC patients with FMF also required biologic therapy earlier, whereas disease outcomes in CD appeared unaffected by FMF status (40).

Studies on *MEFV* variants in IBD have yielded variable results. Some have reported that *MEFV* gene mutations are more frequent in IBD patients compared to control groups, though these findings lack consistency (41, 42). For example, Yurtcu et al. (2009) compared 47 Turkish IBD patients with 25 controls and found *MEFV* mutation frequencies of 19.1% in IBD patients versus 12% in controls, implying that *MEFV* variants may not play a significant role in IBD susceptibility (39). Similarly, Karban et al. (2005) reported that *MEFV* mutations are not considered major risk factors for CD (38). Additionally, no significant differences in *MEFV* mutation prevalence were observed among CD patients with varying NOD2/CARD15 genotypes, and no clinical differences were reported between carriers and noncarriers of *MEFV* mutations (38). Nevertheless, specific *MEFV* variants have been implicated in IBD pathogenesis. One notable variant, E148Q, has been associated with perianal disease, commonly found in CD patients. In fact, it was shown that 21.2% of CD patients with perianal disease were E148Q carriers, compared to only 6.7% of those without perianal disease, suggesting a potential role for E148Q in CD pathogenesis independent of NOD2/CARD15 mutations (38). Another significant *MEFV* variant reported in the literature is the M694V variant. While a recent study identified E148Q as the most common variants among patients with UC and CD, and M694V as the most prevalent among patients with IC (43), other numerous studies seem to agree that M694V variant is the most frequent mutation among IBD patients (44–46). For instance, the screening of FMF-associated *MEFV* variants in Turkish children with IBD revealed that 70% of patients with coexisting FMF and IBD carried the M694V mutation, making it the most common mutation in this group (46). Similarly, another study showed that, among 53 Turkish children suffering from IBD, FMF was diagnosed in 14 patients, predominately carrying the homozygous M694V mutation (45). Notably, FMF was more prevalent in UC patients (78.6%) than in CD patients (21.4%) (45). The same group of researchers found that *MEFV* gene variants were present in all 12 IBD patients studied, with variant frequencies of 41.7% for M694V, 25% for M680I, 25% for K695R, and 8.3% for E148Q (44). This was the first study to establish an association between the K695R mutation and IBD (44). Comparable findings have been reported by other researchers from Turkey and Armenia (47, 48).

The impact of *MEFV* variants on IBD severity remains controversial. Some studies suggest that FMF-associated *MEFV* mutations are linked to a more aggressive IBD phenotype, while others refute this association. For instance, Sahin et al. (2021) reported that UC patients carrying M694V or E148Q variants required surgery more frequently and experienced more severe disease than CD patients (49). However, these findings may be biased, as CD patients in the study had less severe inflammatory profiles, did not require surgery, and mostly lacked *MEFV* mutations (49). Similar observations have been reported in studies and case reports from Israel (40), Armenia (48), and Turkey (50). These reports highlight that IBD patients with concomitant FMF tend to experience a more severe disease course, and their symptoms often persist despite standard IBD treatments. Notably, treatment with colchicine has been shown to induce remission of both conditions, underscoring the importance of early screening for *MEFV* variants to facilitate appropriate disease management (48, 50). Conversely, other studies from Turkey and Israel suggest that disease activity indices are independent of *MEFV* mutations (43, 51, 52). Nonetheless, these studies report that extraintestinal manifestations, including arthritis, arthralgia, edema, myalgia, sacroiliitis, and pyoderma gangrenosum, were more frequent in individuals with *MEFV* mutations (43, 51, 52). Furthermore, some manifestations, like amyloidosis, may worsen as a result of the synergistic relationship between FMF and IBD (52). Even such findings remain debated, with certain Turkish studies failing to establish a clear association between *MEFV* mutations and these manifestations (39, 49).

In summary, studies investigating the association between FMF and IBD suggest that M694V and E148Q are the most common *MEFV* variants in IBD patients. However, whether these variants significantly influence IBD severity and extraintestinal manifestations remains controversial. Due to the inconsistent findings, large, multiethnic studies are needed to better understand the role of *MEFV* mutations in IBD pathogenesis and clinical outcomes.

#### 3.1.2 *FMF and systemic* lupus erythematosus

Systemic lupus erythematosus (SLE) is a chronic autoimmune inflammatory disease characterized by the production of autoantibodies directed against multiple organ systems, including the skin, kidneys, joints, and central nervous system (CNS), leading to tissue damage (53). Although its pathogenesis is not yet fully elucidated, SLE is thought to arise from a complex interplay of genetic predisposition, environmental triggers, hormonal influences, and immunological mechanisms (54). The main features of SLE include polyserositis which manifests as pericarditis, pleuritis, and peritonitis, dysregulation of both the innate and adaptive immune systems, and the generation of abnormal autoantibodies such as anti-double-stranded DNA (anti-dsDNA), anti-Smith (anti-Sm), anti-ribonucleoprotein (anti-RNP), anti-Sjögren’s syndrome antigen A (anti-Ro/SSA), anti-Sjögren’s syndrome antigen B (anti-La/SSB), and anti-phospholipid antibodies (55, 56). These autoantibodies lead to the formation of immune complexes that deposit in tissues. These deposits activate the immune system, triggering the recruitment of monocytes and macrophages and leading to the production of pro-inflammatory cytokines such as TNF (tumor necrosis factor), IL-1, IL-6, and IL-18. Additionally, type I interferons and immunoregulatory cytokines like IL-10 and B-cell activating factor (BAFF) — a member of the TNF family — are involved (57). These cytokines promote B-cell activation and sustained autoantibody production, thereby perpetuating the autoimmune response and contributing to ongoing tissue damage (57).

SLE shares numerous overlapping symptoms with FMF, including fever, fatigue, arthritis, pericarditis, pleuritis, peritonitis, rash, and both musculoskeletal and renal involvement (58, 59). Case studies of patients with both SLE and FMF have shown that fever, abdominal pain, and joint pain are the most frequent FMF symptoms, and these are often successfully managed with colchicine treatment (60–65).

Given the shared clinical features and the involvement of inflammasomes and inflammatory pathways in both diseases, researchers have explored the potential link between FMF and SLE. For instance, a study conducted in South Asia found that SLE patients had a higher prevalence of FMF compared to the non-SLE control group (1.29% vs. 0.79%) (66). Similarly, an Israeli cohort study reported a 0.68% prevalence of FMF among SLE patients, compared to only 0.21% in individuals without SLE (67). However, conflicting findings have emerged from studies in Turkey and Israel, where the frequency of *MEFV* variants in SLE patients was reported to be similar to or even lower than that in healthy controls (58, 68, 69). Additionally, in the study by Ozen and Bakkaloglu (2005), many of the FMF patients included had other inflammatory diseases, but none had SLE. To explain these findings, several researchers from Turkey and Israel proposed a notable concept: a “protective” association between FMF and SLE, suggesting that the presence of FMF-related mutations might actually confer some resistance to developing SLE (70, 71). This association may be attributed to the lack of anti-serum amyloid P (SAP) antibodies. SAP is a protein that belongs to the pentraxin family and is involved in removing nuclear ligands from necrotic and apoptotic cells (72). It has been demonstrated recently that elevated levels of anti-SAP antibodies are linked to active SLE; nevertheless they are not detected in patients with FMF or in patients who have both SLE and FMF (73). Therefore, it is hypothesized that the lack of anti-SAP antibodies in patients with FMF and SLE allows SAP to function normally, leading to a quicker clearance of chromatin and other apoptotic products, thereby promoting a milder SLE phenotype (70). Another molecule that may contribute to this protective association is C-reactive protein (CRP), a member of the pentraxin family and a commonly measured acute phase reactant (71). These molecules are known to bind accessible small nuclear ribonucleoprotein (snRNP) particles and play a significant role in the clearance of apoptotic debris (71). While research suggests that decreased basal CRP expression contributes to the development of SLE (74), CRP concentrations are elevated in FMF patients (71). Therefore, both SAP and CRP may exert protective effects against SLE susceptibility in individuals with FMF.

To investigate the role of *MEFV* variants in organ involvement among SLE patients, Shinar et al. (2012) compared the clinical phenotypes of SLE patients with and without *MEFV* mutations. Their findings suggested that the presence of an *MEFV* variant may influence the SLE phenotype, with carriers displaying a higher prevalence of inflammatory manifestations but a reduced risk of developing renal disease, potentially due to the protective effects of CRP and SAP. This distinction underscores the difference between autoinflammation, which underlies FMF, and autoimmunity, which plays a central role in the development of SLE nephritis (58).

Regarding FMF attacks in SLE patients, studies have found that they tend to be milder, shorter in duration, and associated with lower fever spikes compared to FMF patients without SLE (75, 76). However, in terms of frequency, controversial findings were noted, with some reports indicating a higher FMF attacks prevalence in SLE patients, while others stating that FMF attacks in SLE patients are less common than FMF patients without SLE (75, 76).

In conclusion, although a few studies indicate a higher prevalence of *MEFV* mutations among SLE patients, FMF may confer a protective effect against SLE via mechanisms involving CRP and SAP, which could account for the milder SLE symptoms observed in patients with both conditions. This hypothesis warrants further investigation.

#### 3.1.3 FMF and multiple sclerosis

Multiple sclerosis (MS) is the most common chronic inflammatory demyelinating disease, affecting the CNS. It is characterized by episodes of neurological impairment that can be fully or partially reversible, typically lasting from a few days to several weeks (77). The most common clinical manifestations include ataxia resulting from cerebellar lesions, diplopia caused by brainstem involvement, limb weakness or sensory deficits due to transverse myelitis, and monocular visual loss associated with optic neuritis (77). Many affected individuals experience a progressive clinical course after approximately 10–20 years, ultimately leading to cognitive and mobility impairments (77). A central element in MS pathophysiology is the immune system, involving both innate and adaptive responses. Notably, the disease heavily depends on the adaptive immune system, primarily involving T cells and B cells (78). Furthermore, several genetic factors, particularly within the Human Leukocyte Antigen (HLA) class II region, are associated with an increased risk of developing MS, although no single gene has been identified as the definitive cause (79). Additionally, environmental factors such as viral infections, vitamin D deficiency, and alterations in gut microbiota may interact with genetic predispositions, contributing to the development of MS (80–82). Interestingly, both FMF and MS share common underlying mechanisms, including dysregulation of the innate immune system and a genetic predisposition. Specifically, HLA-related polymorphisms—well-known risk factors for MS—have also been linked to an increased risk of FMF and are considered potential modifier genes for these conditions (83). In fact, numerous case studies have reported the co-occurrence of FMF and MS symptoms, which has sparked further interest in exploring the potential connection between these two diseases (84–91).

At the molecular level, the frequency of *MEFV* variants was found to be higher in MS patients compared to healthy individuals. Several studies conducted in Turkey have identified the M694V mutation as the most common among MS patients relative to controls (86, 92–94). Similar findings have been reported in Israeli and northern European Caucasian MS populations, where the presence of *MEFV* variants, particularly M694V and E148Q, suggests that these mutations may act as potential modifier genes or risk factors for MS (95, 96). Moreover, research on pediatric MS patients indicated that 37.9% carried at least one heterozygous mutation in the *TNFRSF1A* gene, associated with TNF receptor-associated periodic fever syndrome, TRAPS, and/or *MEFV* gene. This rate was significantly higher than that observed in adult MS patients, suggesting a potential role in the pathogenesis of MS during childhood (97). However, such as finding was not observed in other studies from Turkey, which reported no significant difference in *MEFV* mutation frequencies between MS patients and healthy controls (98, 99).

Several theories have been proposed to elucidate the relationship between FMF and MS. The prevailing hypothesis suggests that mutations in the *MEFV* gene along with the production of proinflammatory cytokines, such as IL-1, IFN-γ, and TNF-α, may overwhelm monocytes, impairing their ability to suppress the inflammatory response. This could result in increased secretion of oligotoxic and neurotoxic compounds and in decreased activity of microglial cells in MS lesions (58, 99, 100). However, in the study by Elhani et al. (2021), patients receiving colchicine exhibited higher MS incidences in the absence of peripheral inflammation, which challenges the prevailing theory proposing that the link between FMF and MS is mainly driven by inflammation (98). An alternative hypothesis proposes a potential role of non-inflammatory factors in FMF patients presenting with MS. In fact, rather than being solely driven by inflammation, demyelination in MS is thought to occur prior to T cell infiltration into tissues (101). Consequently, it is suggested that additional mechanisms, such as endothelial dysfunction and vasculitis in FMF, may contribute to disruption of the blood brain barrier, thereby initiating MS lesion formation (101). Furthermore, the elevated body temperature experienced during FMF attacks might impair the function of myelin and mitochondrial proteins, potentially promoting disease progression (101).

The influence of *MEFV* mutations on MS disease progression has been investigated in many studies yielding inconsistent results. Shinar et al. (2003) reported similar frequencies of *MEFV* mutations among primary progressive (PP) and relapsing–remitting (RR) MS patients within European Jewish (Ashkenazi), Iraqi Jewish, and North-African Jewish populations. These patients also experienced increased disability, higher relapse rate, and lower attack rate. However, the researchers found that these disease progression characteristics varied between different ethnicities. For example, Iraqi and Jewish M694V carriers demonstrated rapid disease deterioration as opposed to Ashkenazi carriers that showed no significant variation (102). In fact, gene variants, namely, delta 32 CCR5, prevalent among Ashkenazi Jews, may inhibit the expression of *MEFV* mutations, accounting for a milder MS course (103). Moreover, in the Ashkenazi group, the consensus HLA type II haplotype for individuals with MS, DRB1*1501, DQA1*0102, and DQB1*0602, is more common than in the Iraqi Jewish cohort, further increasing the genetic heterogeneity between the two cohorts and potentially affecting the rate of MS progression (102, 104). In accordance with Shinar et al.’s (2003) findings, Unal and colleagues found that the annual relapse rate was slightly higher among RR MS patients carrying *MEFV* mutations as compared to non-carrier RR MS patients, suggesting that *MEFV* mutations may confer a more progressive disease course. Nevertheless, these findings were contradicted by recent studies showing that MS patients with *MEFV* mutations had similar disease course, clinical features, severity, response to therapy, and progression to patients with no *MEFV* mutations (95, 96, 99). The only exception was observed in patients with K695R mutations who exhibited a more severe disease course with higher progression rates, leading to brain atrophy and cognitive deficits in some severe cases (95). Besides K695R mutation carriers, another study reported that patients homozygous for M694V mutation experienced a more progressive MS course (96).

To summarize, the relationship between MS and FMF appears to be mediated by both inflammatory and non-inflammatory mechanisms, with different *MEFV* variants exerting distinct effects on MS disease progression. While some mutations may exacerbate MS severity, the overall impact remains inconsistent across studies, highlighting the need for further research to elucidate the precise role of *MEFV* variations in MS pathogenesis and progression.

#### 3.1.4 FMF and rheumatoid arthritis

Arthritis can be defined as an acute or persistent inflammation of the joints (105). Several symptoms, including pain, stiffness, reduced range of motion, and joint abnormalities, can be attributed to this disease (105). Arthritis can exist in various forms; the most prevalent type is osteoarthritis, known as degenerative non-inflammatory arthritis (105). However, inflammation-related arthritis is also common and can arise from various causes, including autoimmune conditions such as rheumatoid arthritis (RA), psoriatic arthritis, and ankylosing spondylitis; infectious etiologies like septic arthritis and Lyme’s arthritis; and crystal deposition diseases such as gout, pseudogout, and basic calcium phosphate disease (105).

Several key factors contribute to the pathogenesis of RA. They include environmental factors (such as infections, smoking, and obesity), genetic predispositions (notably HLA alleles like HLA-DRB1 and HLA-DR4), and autoimmune responses (106, 107). The autoimmune nature of RA results in the overactivity of both T and B cells in affected patients. This process involves cytokine release, such as IL-12 and IL-18, and intercellular communication that activates T cells near macrophages, thereby amplifying B-cell cytokine production even in the absence of direct cell contact (108).

The *MEFV* gene is located within a susceptibility region for RA on chromosome 16p13-q12.2, prompting researchers to explore the potential relationship between FMF and RA (109). Indeed, several case studies have reported the co-occurrence of FMF and RA (110–114). For example, in Turkey, Israel, and Japan the frequency of *MEFV* variants in RA patients was shown to be comparable to that in the general population, with E148Q being the most prevalent variant in RA patients (115–118). On the other hand, an Israeli study involving 98 RA patients and 100 healthy controls found that carriers of *MEFV* mutations tend to have a later disease onset, suggesting that these variants may have a minimal or potentially protective role in predisposing individuals to RA (118). Similar findings were reported in Turkish and Japanese cohorts (115–117): in the Japanese research, no significant association was noted between *MEFV* variants and either RA onset or RA-related amyloidosis (117). Similarly, studies conducted by Inanir et al. (2013) and Koca et al. (2010) in Turkey identified only a statistically significant difference in the number of deformed joints among patients, but these findings were insufficient to conclusively determine whether RA patients with *MEFV* variants experience more severe clinical outcomes (115, 116). Interestingly, a recent Moroccan study revealed contrasting results, showing a higher *MEFV* mutation rate of 24% in RA patients compared to 2% in the control group (119). These findings align with those reported by Kalyoncu and colleagues, who demonstrated that the incidence of RA is significantly higher in asymptomatic variant carrier parents of FMF patients compared to healthy subjects (120).

Regardless of whether *MEFV* variants are considered predisposing factors for the development of RA, evidence suggests that they may influence disease severity (116, 118–120). For example, compared to healthy subjects, asymptomatic RA patients carrying *MEFV* mutations have been observed to experience a minimum of four febrile episodes per year, along with increased incidences of arthralgia, acute rheumatic fever, and more severe RA symptoms (120). This might be attributed to the elevated baseline levels of inflammation in patients carrying *MEFV* variants, which potentially increases their susceptibility to complications (120–122).

In an attempt to understand how *MEFV* mutations affect RA severity, several theories emerged. On one hand, some authors attributed this association to the formerly reported relationship between some *HLA-DR/DQ* alleles that are directly linked to RA severity and *MEFV* variants in FMF patients (123, 124). Notably, the *HLA-DR4* allele was found at a higher frequency in FMF patients compared to their family members and healthy controls. Additionally, specific mutations such as M694V were associated with *HLA DR3*, *HLA DR11/5, HLA DR 13/6*, *HLA DQ6/1*, *HLA DQ7/3*, and *HLA DQ8/3* alleles; M680I with *HLA DR7* and *HLA DQ2* alleles; and V726A *HLA DQ6/1* (123). On the other hand, other researchers relate this association to the role of the *MEFV* gene in the deregulated inflammatory process of RA. Particularly, the increased IL-1β production in *MEFV* carriers may contribute to the severity of RA symptoms in these patients (118, 119).

In conclusion, *MEFV* mutations appear to influence the severity and progression of RA rather than being directly involved in the development of the disease in individuals.

#### 3.1.5 FMF and Sjögren’s syndrome

Sjögren’s syndrome (SS) is a chronic autoimmune disease, characterized by the infiltration of inflammatory CD4^+^ T cells and cytokines into exocrine glands, including salivary and lacrimal glands, leading to neurological symptoms, joint discomfort, fever, and dry eyes and mouth, termed the sicca syndrome (125). Particularly in its early phase, SS may affect other organs and systems, resulting in a variety of clinical manifestations, namely, exocrine glandular and extra-glandular presentations (126). SS can either appear as a primary disease that is unrelated to other illnesses or secondary to other inflammatory diseases such as SLE, RA, systemic sclerosis, polymyositis, and FMF (126). The coexistence of SS and FMF is of particular interest because both diseases were reported to be together associated with other diseases including hepatitis, ankylosing spondylitis, and mixed connective tissue disease (125, 127, 128).

Previous case studies have documented patients diagnosed with both FMF and SS. The first reported case was a 42-year-old man who had a 20-year history of FMF and complained of dry mouth and eyes, which was later associated with SS (129). Analysis of his genetic profile revealed a compound heterozygosity for the M694V mutation (129). However, the authors suggested that this association is likely coincidental and does not necessarily indicate a true relationship between SS and FMF; therefore, further studies are needed to better elucidate the nature of this potential link (129). Similarly, another case study described a 42-year-old Japanese woman suffering from SS (130). Due to her recurrent attacks and homozygosity for the M694I mutation, she was diagnosed with FMF and immediately started colchicine treatment (130). Her previously elevated IL-18 levels were efficiently reduced upon colchicine administration, suggesting that the FMF-related dysregulated IL-18 production and chronic inflammation may be attributed to the occurrence of SS (130).

In conclusion, the coexistence of FMF and SS appears to be rare and underreported, and its underlying mechanisms remain poorly understood, warranting further research.

#### 3.1.6 FMF and celiac disease

Celiac disease (CD) is a life-long gluten-sensitive autoimmune disease of the small intestine affecting genetically susceptible individuals worldwide (131). The disease is characterized by gastrointestinal-related symptoms such as diarrhea, steatorrhea, and weight loss due to malabsorption (131). The variable clinical picture of CD is attributed to genetic and immunological factors, with the age of onset, extent of mucosal injury, dietary habits, and gender influencing the clinical presentation of the disease (131). CD diagnosis is based on the presence of a predisposing genetic factor, HLA DQ2/8, along with positive biopsy and serological antibodies upon the consumption of a gluten-containing diet (131). CD is triggered by the ingestion of gliadin and other related prolamins. Gliadin, a major component of gluten, is a 30 kDa alcohol-soluble protein rich in glutamine and proline and resistant to gastrointestinal enzymes (132). Upon ingestion, gliadin peptides are transported from the lumen of the gut to the lamina propria of the small intestine where tissue transglutaminase 2 catalyzes their deamidation, converting glutamine residues to negatively charged glutamic acid (132). Subsequently, modified gliadin peptides are recognized by antigen-presenting cells (APC) and presented on their surface in association with HLA-DQ2 or HLA-DQ8 which, unlike other HLA-DQ molecules, have a high binding affinity to the deaminated form of gliadin (132, 133). In fact, patients homozygous for HLA-DQ2/8 are more susceptible to develop CD than their heterozygous counterparts (134). Following gliadin-HLA complex presentation, CD4+ T cells recognize this complex leading to T cell activation, inflammatory response stimulation, and cytokine release, all of which cause intestinal mucosa damage and villous atrophy (133).

CD and FMF share a common inflammatory etiology, prompting several case studies to investigate their co-existence (135–137). However, diagnosing CD in FMF patients (or vice versa) is challenging due to their overlapping clinical features such as abdominal pain and diarrhea (138). Although the exact nature of the association remains unclear, it is believed that the amplified ongoing inflammation in FMF activates intestinal mucosal immune cells, leading to intestinal lesions and contributing to CD pathogenesis (139, 140).

The prevalence of CD was evaluated in two separate studies involving children with FMF and children with CD. In the first study, 50 FMF children were tested for anti-gliadin antibodies (AGA) (IgA/IgG) and anti-endomysium antibodies (EMA) (IgA/IgG), while 17 children with CD were assessed for *MEFV* variants, clinical features, and laboratory findings (141). None of the FMF patients were diagnosed with CD and none of the CD children showed consistent FMF clinical manifestations despite three CD children being heterozygous for the E148Q and one child heterozygous for M680I (141). The researchers concluded that *MEFV* variants were similarly prevalent in CD patients and the general population, indicating no association between CD and FMF (141). In a second study, Işikay et al. (2015) evaluated 112 FMF patients and 32 healthy controls by measuring serum tissue transglutaminase IgA (tTG IgA). Four participants—three FMF patients and one control—had positive tTG IgA levels and underwent gastroscopy, which confirmed CD diagnosis. HLA typing revealed HLA-DQ8 positivity in one FMF patient and HLA-DQ2 positivity in the other three confirmed CD cases, further suggesting no significant link between CD and FMF (142). However, both studies faced limitations, including small sample sizes and the absence of the ESPGHAN (European Society for Paediatric Gastroenterology Hepatology and Nutrition) criteria for accurate CD diagnosis. To address these issues, Sahin et al. (2017) conducted a larger longitudinal study from October 2015 to March 2016, involving 303 FMF children, using ESPGHAN criteria for diagnosis. Among them, nine tested positive for tTG IgA, but only one exhibited high EMA IgA levels. Gastro-duodenoscopy in these patients showed no evidence of CD, leading the researchers to conclude that CD is not prevalent among children with FMF and that there is no significant association between the two conditions (138). Overall, these studies suggest that CD is unlikely to be a common complication in pediatric FMF.

In summary, existing research does not support a strong clinical relationship between CD and FMF. The main limitations of previous studies include small sample sizes and inconsistent application of diagnostic criteria. Future research should aim for larger, more standardized investigations to explore potential genetic or inflammatory links that may exist between CD and FMF.

#### 3.1.7 FMF and psoriasis

Psoriasis is an autoimmune disease characterized by immune cell infiltration into the dermis, epidermal hyperplasia, and a complex pathogenesis involving interactions between immune cells, keratinocytes, and other skin-resident cells (143). Over the past two decades, research has identified immune cells as the primary drivers of psoriasis, with keratinocytes acting as mediators of immune dysregulation in the disease process. The IL-23/IL-17 pathogenic axis plays a central role in psoriasis pathogenesis (143). Once T helper 1(Th1) and Th17 cells are activated, plasmacytoid dendritic cells stimulate the maturation of myeloid dendritic cells, leading to the production of inflammatory cytokines such as TNF-α, IL-12, and IL-23, which are crucial in orchestrating the skin’s immune response (143). Particularly, TNF-α, IL-17, IL-21, and IL-22 stimulate keratinocytes, which in turn amplify inflammation. Among these, IL-17 is particularly significant for its role in promoting the production of antimicrobial peptides, cytokines, and chemokines by keratinocytes (143). This keratinocyte activation creates a feedback loop that sustains chronic inflammation, leading to hallmark features of psoriasis, such as epidermal thickening and plaque formation.

Both FMF and psoriasis exhibit cutaneous manifestations and share underlying pathogenic features, such as dysregulated proinflammatory cytokine release and amplified systemic inflammation. The frequency of psoriasis among patients with FMF has been assessed in a cohort of 351 FMF patients (177 adults and 174 children) (144). Interestingly, among the 351 patients, psoriasis was identified in 13 FMF patients, consisting of 11 adults and 2 children. Moreover, 12.4% of adult FMF patients and 5.2% of juvenile patients had psoriasis (144). Therefore, researchers concluded that psoriasis may be occurring at a higher rate in FMF patients as compared to the general population, with adult patients being at greater risk than juvenile patients since the onset of psoriasis typically peaks around age 20, with a second peak observed between ages 50 and 60 (144, 145). The increased prevalence of psoriasis in FMF patients is thought to be linked to the elevated levels of active IL-1, which promotes Th17 cell activation and stimulates keratinocytes, contributing to psoriatic pathology (144). Supporting this, Ashida et al. (2016) reported an abundance of Th17 cells in the upper dermis of psoriasis-like lesions in a patient with FMF (146). Similarly, the occurrence of psoriasis among FMF patients has been examined by Barut et al. (2014) in a group of 202 FMF patients. In accordance with Erden et al. (2018) findings, psoriasis was observed in 41 patients, accounting for 20.3% of the cohort (147). Given these findings, it is important for clinicians to thoroughly evaluate psoriasis lesions and inquire about family history in FMF patients. However, further research, particularly prospective multicenter studies, is necessary to better understand the incidence and underlying mechanisms of psoriasis in the FMF population (144).

#### 3.1.8 FMF and Hashimoto’s thyroiditis

Hashimoto’s thyroiditis (HT) is an autoimmune thyroid disease characterized by the destruction of thyroid cells through both cell- and antibody-mediated immune mechanisms (148). It has been proposed that shared inflammatory pathways, particularly cytokine dysregulation in FMF, may provoke autoimmune responses and predispose to thyroid autoimmunity. Case reports support this possible link; for instance, Gulcan et al. (2009) described a 21-year-old woman with coexisting FMF and HT, suggesting that cytokine expression in FMF may act as a trigger for thyroid autoimmunity (149, 150). Similarly, a case of a 10-year-old girl with FMF carrying a heterozygous E148Q *MEFV* mutation who later developed HT has been reported. Her thyroiditis was confirmed by high anti-thyroid antibody titers and ultrasound results, and treatment with levothyroxine led to resolution of symptoms, further supporting a potential association between FMF and autoimmune thyroid disease (149). Moreover, Dikbas et al. (2013) found that, although not statistically significant in his small cohort, thyroid autoimmunity was more common in FMF patients compared to healthy controls, with significantly higher levels of thyroid autoantibodies observed in the FMF group (151).

However, more recent data have challenged this association. In a Turkish cohort of 133 pediatric FMF patients and 70 healthy controls, serum levels of thyroid-stimulating hormone, free thyroxine, and thyroid autoantibodies were comparable between groups, suggesting no increased prevalence of thyroid dysfunction or autoimmunity in FMF (152). In agreement with the study by Turan et al. (2021), a retrospective analysis of 421 French adult FMF patients documented a prevalence of HT of 1.6%, which is within the estimated range in the general population (1.35–8.4%) (153). These findings imply that regular screening of thyroid function and autoantibody levels may be not necessary in FMF patients in the absence of clinical symptoms or family history (152). Overall, the available data about the association between FMF and HT are limited in literature, mostly observational, and sometimes inconsistent, which underscores the need for further investigation.

### 3.2 FMF and metabolic diseases

#### 3.2.1 FMF and Type 1 diabetes mellitus

Autoimmune diabetes, or type 1 diabetes mellitus (T1DM), is a chronic inflammatory disease characterized by an autoimmune reaction against pancreatic beta cells, hence leading to insufficient insulin production and hyperglycemia. Th17 cells are central to T1DM pathogenesis, with proinflammatory cytokines such as IL-1 and IL-6 promoting their differentiation (154). In fact, elevated serum levels of TNF-α, IL-1, and IL-6 have been observed in diabetic patients compared to healthy controls, further supporting their role in disease development (155, 156). Moreover, recent studies on mice have also implicated NLRP3 inflammasome activation and excessive IL-1 production in the development of T1DM (157). Considering that FMF is characterized by increased IL-1 levels, it is hypothesized that this proinflammatory milieu may contribute to the development of T1DM in genetically susceptible individuals. Thus, the immune dysregulation seen in FMF could potentially predispose individuals to autoimmune mechanisms underlying T1DM.

While T1DM is frequently associated with other autoimmune disorders such as Addison’s disease, autoimmune thyroid disease (ATD), and celiac disease, the co-occurrence with FMF is rare (158). Only a few reports have documented this association, making it a relatively novel observation in the medical literature. For instance, Atabek et al. (2006) described a 9-year-old girl who had T1Dm and experienced recurrent stomach pain since childhood (159). Her symptoms persisted inexplicably despite a thorough medical evaluation until a family history of FMF prompted genetic testing, which revealed that she and her parents carried the M680I mutation (159). Although the patient was only heterozygous, colchicine medication greatly reduced her symptoms. Thereby, the patient’s persistent stomach pain, which was previously attributed to unknown reasons, may have been caused by undiagnosed FMF, which raises the possibility of a connection between FMF and T1DM (159). Similarly, Baş et al. (2009) reported a case of a 10.5-year-old boy who initially presented with T1DM, autoimmune thyroid disease, and celiac disease, and subsequently developed recurrent febrile attacks accompanied by abdominal and chest pain (135). Molecular analysis confirmed an M694I/V726A compound heterozygous genotype, and colchicine therapy alleviated the symptoms significantly. In another case, Gicchino and colleagues reported a 13-year-old boy who had been diagnosed with T1DM at age four and later presented with recurrent fever, abdominal pain, chest pain, and arthralgia. Genetic analysis revealed a homozygous E148Q variation, confirming FMF, and treatment with colchicine resulted in significant symptom relief (158). Collectively, these cases highlight the need to consider FMF in the differential diagnosis of unexplained febrile or inflammatory symptoms in patients with T1DM.

To investigate the potential association between T1DM and FMF, Anwar et al. (2015) conducted a study involving 45 Egyptian children with T1DM and 41 healthy controls to screen for *MEFV* mutations. The results showed an *MEFV* variant frequency of 42.2% in diabetic patients and 34.1% in healthy subjects, with no statistically significant difference. Notably, heterozygosity was observed in 31.1% of diabetic patients, most commonly for E148Q, followed by A744S, V726A, M680I (G/C), and P369S, with 11.1% being compound heterozygotes. The elevated variant carrier rate in both groups is likely reflective of the high consanguinity within the studied population (160). Although no significant difference in *MEFV* mutation frequency was found between diabetic and non-diabetic children, these findings emphasize the importance of ongoing clinical monitoring. FMF should be considered in T1DM patients presenting with recurrent, self-limiting febrile episodes, abdominal or chest pain, or arthritis. Moreover, the shared proinflammatory cytokine pathways between FMF and T1DM suggest potential mechanistic links and may provide opportunities for targeted therapeutic approaches.

#### 3.2.2 FMF and metabolic syndrome

Metabolic syndrome (MetS) is a cluster of metabolic abnormalities, including insulin resistance (IR), hypertension, dyslipidemia, and Type 2 diabetes mellitus (T2DM), which is five times more likely to occur in MetS patients compared to healthy individuals (161). The role of chronic inflammation in MetS pathogenesis has been well established, with early studies identifying TNF-α as a key driver of IR and human T2DM through suppression of the insulin receptor substrate-1 (IRS-1) signaling pathway. TNF-α also activates NF-κB and activator protein 1 (AP-1), amplifying the inflammatory cascade and further disrupting glucose metabolism (162, 163). Alongside TNF-α, IL-6 plays a critical role in promoting systemic inflammation and insulin resistance, as demonstrated by Stenlöf et al. (2003) and Xu et al. (2017) (164, 165). Additionally, the infiltration of immune cells, particularly macrophages, into adipose tissue fosters a chronic low-grade inflammatory state that exacerbates metabolic dysfunction (166).

Given the central role of inflammation in MetS, researchers have explored whether genetic predispositions, specifically *MEFV* gene mutations, contribute to metabolic dysfunction (167). Balkarli et al. (2016) reported a significantly higher prevalence of the R202Q *MEFV* mutation in 50 MetS patients compared to 50 healthy controls; however, this mutation did not appear to directly influence clinical markers of MetS severity (168). Likewise, a cross-sectional study involving 154 FMF patients (66 males and 88 females) and 154 age- and sex-matched controls showed that the frequency of MetS among FMF patients was higher (42.90%) compared to controls (28.57%) (169). Additionally, serum uric acid levels were elevated in FMF patients, leading researchers to suggest that hyperuricemia could serve as a reliable biochemical marker for MetS in FMF patients, aiding in early diagnosis and intervention (169). More recently, a 2024 case-control study investigated metabolic syndrome components in Egyptian children with FMF during attack-free periods (170). Although none of the children met the full criteria for MetS, they exhibited significantly higher insulin resistance levels compared to healthy controls, indicating that chronic subclinical inflammation may predispose these children to develop MetS later in life (170).

Collectively, these findings underscore the intricate interplay between genetic susceptibility, inflammation, and metabolic disturbances in FMF patients. While *MEFV* mutations and inflammatory cytokines such as IL-1β may create a proinflammatory environment that promotes insulin resistance, additional factors such as adipose tissue dysfunction and hyperuricemia likely contribute to metabolic deterioration. Given these insights, further research is essential to elucidate the precise mechanisms underlying the FMF-MetS connection, which may lead to targeted therapeutic strategies to mitigate metabolic risk in FMF patients.

#### 3.2.3 FMF, amyloidosis, and goiter

Amyloidosis is a disorder characterized by the accumulation of proteins into amyloid fibrils and formation of a β-pleated sheet structure in various human tissues and organs (171). The key mechanism that underlies amyloid pathology is the protein’s capacity to acquire many conformations, which makes it amyloidogenic (172). In fact, aberrant proteolysis, point mutations, and post-translational modifications such as phosphorylation, oxidation, and glycation may cause proteins to lose or be unable to acquire their physiologic and active fold, leading to the formation of amyloid fibrils which eventually settle in the target organs’ interstitial spaces (172). These extracellular deposits may either precipitate where they were generated, which would cause a localized form of amyloidosis similar to Alzheimer’s disease, or cause systemic amyloidosis (173). Among the systemic amyloidosis is the secondary AA amyloidosis caused by any long-term inflammatory disease through the accumulation of SAA protein. SAA is an acute phase reactant produced by hepatocytes as well as other cells such as macrophages, endothelial cells, and smooth muscle cells and is regulated transcriptionally by proinflammatory cytokines, specifically TNF- α, IL-1β, and IL-6 (174). In addition, SAA has been shown to participate in immunity regulation and inflammation by activating immune cells at the site of inflammation and promoting the release of proinflammatory cytokines and chemokines (175). Thereby, AA amyloidosis may exacerbate a number of chronic inflammatory disorders, including cancer, autoinflammatory diseases, chronic infections, and idiopathic inflammatory diseases (176). FMF, among other diseases, is one of the primary causes of amyloidosis (171). Given the life-threatening nature of FMF, its connection with amyloidosis, more specifically, AA-type amyloidosis, is crucial. While colchicine successfully prevents FMF incidents and amyloidosis from developing, it is still difficult to anticipate the onset of amyloidosis purely from clinical and demographic factors (177). Therefore, previous studies and case reports have examined the correlation between FMF and AA amyloidosis to grasp a better understanding of their relationship.

In 2004, Atagunduz et al. examined the frequencies of three common *MEFV* mutations (M694V, M680I, and V726A) in 37 Turkish FMF patients with amyloidosis (AA-FMF), 35 FMF patients without amyloidosis (non-AA-FMF), 19 patients with secondary amyloidosis unrelated to inflammatory diseases (S-AA), and 185 healthy controls (178). They found that *MEFV* mutations were significantly more prevalent in both AA-FMF (81%) and non-AA-FMF (62.7%) patients compared to healthy controls (4.2%), and in AA-FMF compared to non-AA-FMF patients (178). M694V was the most frequent mutation in both FMF groups (63.5% vs. 51.4%), although allele frequency and homozygosity rates did not differ significantly between them (178). Interestingly, S-AA patients also had a higher rate of *MEFV* mutations than controls, and M694V was the only mutation detected in this group. Overall, these findings indicate that *MEFV* mutations are increased in both FMF and non-FMF-associated secondary amyloidosis, but no clear association exists between M694V and amyloidosis development in FMF (178). In agreement, Tekin et al. (2000) showed that FMF patients lacking M694V are still at risk for amyloidosis and that genetic mutations alone cannot fully account for its development, suggesting the involvement of additional genetic or environmental factors (179).

In contrast, a study from Algeria involving 28 FMF patients with biopsy-confirmed renal AA amyloidosis and 20 matched FMF patients without amyloidosis found a strong genotype–phenotype correlation (180). Sequencing of exon 10 revealed that 87.5% of affected patients carried mutant alleles, with M694I being the most common variant (62.5%), followed by M694V (17.85%), M680I (5.35%), and I692Del (1.78%) (180). Notably, M694I/M694I homozygosity was present in 52% of patients with amyloidosis and was significantly associated with the development of amyloidosis compared to controls (180). Although M694V/M694V was detected only in patients with amyloidosis (11%), this finding was not statistically significant (180). The discrepancies between studies suggest that the contribution of specific *MEFV* variants to amyloidosis risk may vary across populations, potentially reflecting differences in ethnic or regional genetic backgrounds, environmental exposures, or gene-environment interactions.

Another important type of amyloidosis is amyloid goiter, characterized by a gradually growing enlarging thyroid gland that is often asymptomatic initially. Generally, about 30–80% of individuals with secondary amyloidosis and 50% of those with original amyloidosis experience amyloid deposition in the thyroid (181). In FMF, amyloid goiter has been documented in several studies. Özdemir et al. (2001) reported pathological amyloid goiter in 10 of 22 FMF patients, confirmed by biopsy and histological examination. The majority of patients had non-tender thyroid enlargement, with varying degrees of severity, and hypoactive nodules were commonly observed (182). Additional case reports have further supported the association of FMF with amyloid goiter (181, 183–185) Interestingly, some cases of amyloid goiter in FMF have been linked to the E148Q variant (186), which is generally regarded as either a low-penetrance pathogenic variant or a benign polymorphism. While E148Q is usually associated with less severe disease and lower risk of systemic amyloidosis, these findings suggest that even mild *MEFV* variants can contribute to localized amyloid deposition in the thyroid, highlighting the variable phenotypic expression of FMF (186). These cases show the importance of considering amyloid infiltration of the thyroid in FMF patients, even in those with mild *MEFV* variants, to ensure early detection and appropriate management.

#### 3.2.4 FMF and nonalcoholic fatty liver disease

Nonalcoholic fatty liver disease (NAFLD) encompasses a spectrum of liver conditions, ranging from simple steatosis (fatty infiltration of the liver) to nonalcoholic steatohepatitis (NASH), characterized by steatosis with inflammation and hepatocyte necrosis, and eventually cirrhosis (187). The onset of fatty liver can be attributed to multiple factors, including lipid metabolism abnormalities, lipoatrophy, insulin resistance, metabolic syndromes such as obesity and diabetes, certain drugs like steroids, severe weight loss, total parenteral nutrition, and exposure to toxins such as organic solvents (187).

It has been suggested that the development of NASH occurs through a two-step process (188). The first step involves hepatic fat deposition, which exacerbates insulin resistance, while the second involves oxidative stress and fatty acid oxidation in the liver, driven by cytokine-mediated injury, hyperinsulinemia, extracellular matrix alterations, energy imbalance, and immune system changes (188). The pathological progression of NAFLD may follow three interrelated pathways: steatosis, lipotoxicity, and inflammation. Steatosis activates the transcription factor NF-κB, increasing the production of proinflammatory mediators such as IL-6, TNF-α, and IL-1β (189). These cytokines recruit and activate resident hepatic macrophages (Kupffer cells), further mediating inflammation in NASH (189). Additionally, TNF-α and IL-6 contribute to hepatic insulin resistance through upregulation of Suppressor of Cytokine Signaling 3 (SOCS3) (190). The resulting imbalance between pro- and anti-inflammatory cytokines, favoring proinflammatory signaling, promotes recurring or chronic inflammation and lipid accumulation. Given this central role of systemic inflammation and cytokine dysregulation, a co-occurrence between FMF and NAFLD may be expected.

To explore the potential association between FMF and NAFLD, Rimar et al. (2011) examined 27 FMF patients who were referred to a liver clinic because of hepatic function abnormalities persisting for more than 6 months (191). The liver biopsy revealed the presence of NAFLD in 15 patients: 5 with simple steatosis, 3 with NASH, and 7 with NASH-cirrhosis. These findings suggest a potentially underrecognized link between FMF and NAFLD (191). At the molecular level, researchers reported that the homozygous M694V mutation, which was prevalent among the FMF participants (70%), exacerbated the chronic inflammatory state characteristic of FMF, thereby influencing the clinical presentation of NAFLD and promoting its progression to NASH (191). Collectively, these observations suggest that persistent systemic inflammation in FMF may predispose patients to a higher risk of developing severe NAFLD (191). However, Sarkis et al. (2012) documented contrasting results in a cohort of 52 FMF patients and 30 healthy controls, finding that NAFLD was equally frequent in both groups (192). Likewise, a more recent study assessing the prevalence of NAFLD in 54 FMF patients and 54 matched controls detected no significant difference in NAFLD prevalence between the groups (193). Additionally, NAFLD presence was not associated with disease severity, duration, or colchicine dose (193). Overall, the study suggests that FMF patients do not have an increased risk of NAFLD, despite ongoing subclinical inflammation (193).

In summary, although studies on the association between FMF and NAFLD have yielded inconsistent results, chronic or recurrent inflammation in FMF seems to contribute to liver injury in some patients. Therefore, regular monitoring of liver function and colchicine intake are recommended to help prevent hepatic complications.

### 3.3 Synthesis of key interactions between FMF and autoimmune and metabolic diseases

**Figure 2:**
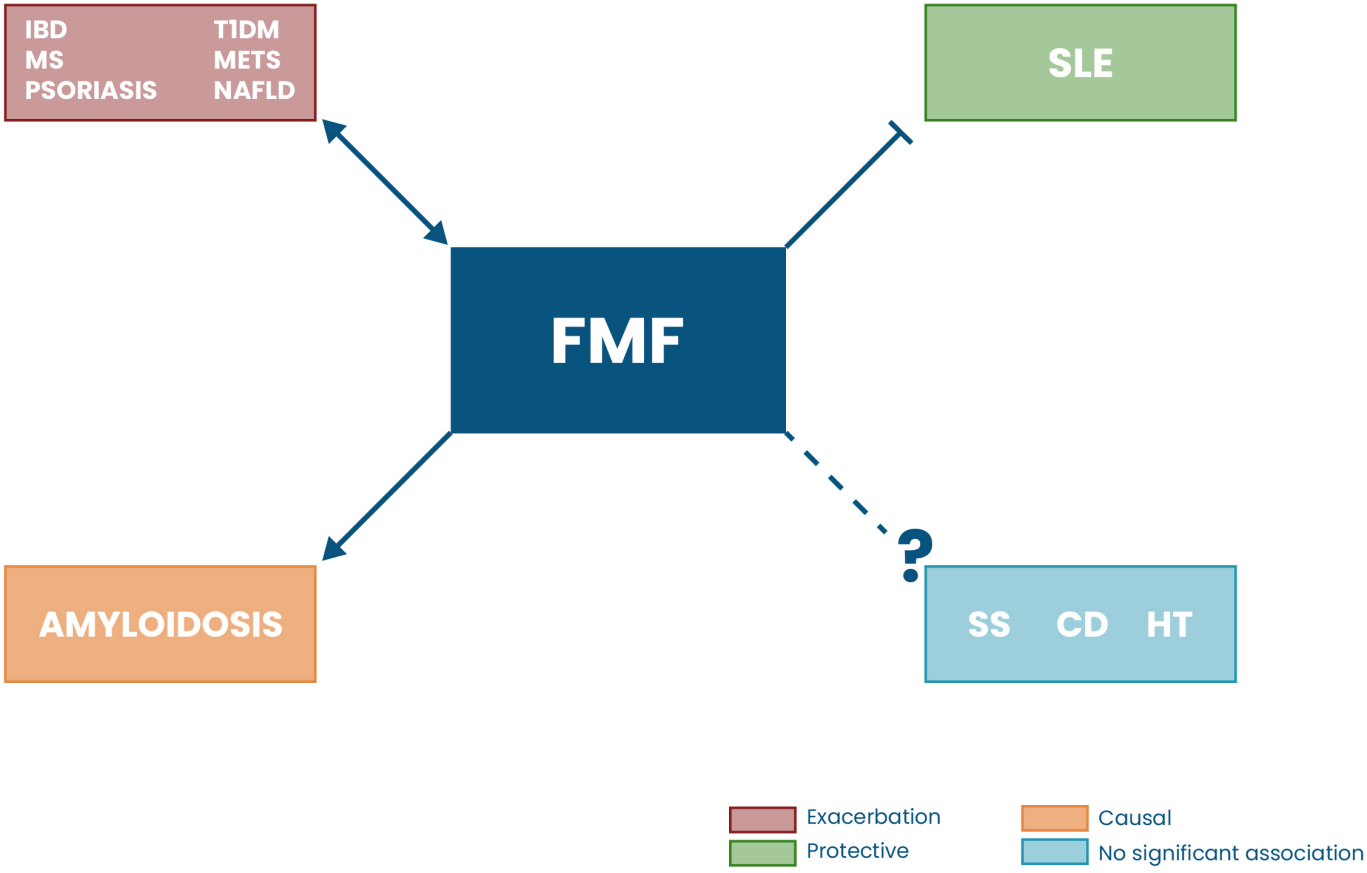
Potential links between FMF and autoimmune and metabolic diseases. FMF exhibits diverse interactions with several immune-mediated and metabolic conditions. Diseases shown in red (inflammatory bowel disease (IBD), multiple sclerosis (MS), psoriasis, type 1 diabetes mellitus (T1DM), metabolic syndrome (MetS), and nonalcoholic fatty liver disease (NAFLD)) represent conditions in which FMF has been associated with exacerbation or increased risk, likely through persistent pyrin inflammasome activation and downstream inflammatory signaling. In green, the association with systemic lupus erythematosus (SLE) is rather protective, where the inflammatory environment in FMF reduce lupus susceptibility. Amyloidosis, shown in orange, represents a causal complication directly resulting from chronic inflammation in FMF. Conditions shown in blue (sjögren’s syndrome (SS), celiac disease (CD), and Hashimoto's thyroiditis (HT)) indicate no significant association, based on available evidence.

The associations between FMF and its comorbidities highlight its role as a broad immunoinflammatory mediator, bridging innate and adaptive immune dysregulation. Through pyrin inflammasome activation, FMF creates a proinflammatory environment that can predispose to, exacerbate, or mimic other disorders (**Figure 2**). Autoimmune conditions such as IBD, RA, MS, and psoriasis demonstrate a bidirectional interaction with FMF: FMF amplifies systemic inflammation, while these diseases, in turn, intensify FMF-related immune activation, resulting in more severe symptoms and disease manifestations. Interestingly, the coexistence of FMF and SLE appears to be protective, as the heightened inflammatory state in FMF mitigates SLE symptoms rather than worsening them. On the other hand, conditions such as SS, CD, and HT show no consistent clinical association with FMF. Amyloidosis represents the most direct and causal complication of FMF, arising from chronic SAA-driven inflammatory deposition, whereas metabolic comorbidities, including T1DM, NAFLD, and MetS, may result from sustained cytokine imbalance, insulin resistance, and inflammasome-mediated tissue inflammation. Collectively, these observations indicate that FMF functions as a systemic inflammatory state capable of influencing the onset, course, and clinical expression of a variety of autoimmune and metabolic disorders, with the magnitude and direction of these interactions dependent on disease-specific pathophysiology.

**Table 1** summarizes the relationship between FMF and co-occurring disorders, namely, autoimmune and metabolic diseases.

**Table 1:** Overview of autoimmune and metabolic diseases associated with FMF: underlying mechanisms, genetic and epidemiological patterns, and clinical outcomes.

Disease	Shared pathogenic mechanism with FMF	Key *MEFV* variants	Epidemiological evidence	Clinical consequences	Type of association
IBD	- Pyrin and NLRP3 inflammasomes dysregulation - Pyrin is linked to autophagy defects, which is implicated in IBD **-** Innate immunity overlap: pyrin and NOD2/CARD15 proteins share structural/functional similarities in cytokine processing and bacterial response	M694V, E148Q, and K695R	- Higher FMF prevalence in IBD (greater frequency in CD vs UC) - FMF is predominately diagnosed prior to IBD - Mutation frequencies are variable across studies	- FMF may worsen UC severity, require earlier biologic therapy, or contribute to certain extraintestinal features - Colchicine may help concurrent symptoms	Exacerbation
SLE	- Both FMF and SLE involve inflammation, fever, arthritis, serositis (pericarditis, pleuritis, peritonitis), and renal/musculoskeletal involvement - Dysregulated inflammasomes and pro-inflammatory cytokines (TNF, IL-1, IL-6, IL-18) - High levels of CRP and SAP in FMF aids in apoptotic debris clearance	M694V and E148Q	- Some cohorts show slightly higher FMF in SLE while others show similar or lower *MEFV* frequency vs controls	- Coexisting FMF often milder FMF attacks and reduce renal involvement in SLE - Colchicine may help concurrent symptoms	Protective
MS	- Dysregulation of innate immunity and pro-inflammatory cytokines (IL-1, TNF-α, IFN-γ) may affect microglial function and lesion development - FMF-related endothelial dysfunction, vasculitis, and febrile episodes may contribute to blood–brain barrier disruption and demyelination - *MEFV* variants may act as modifier genes and HLA class II polymorphisms may interact with *MEFV* mutations	M694V, E148Q, and K695R	- Several cohorts show increased *MEFV* frequency; others show no difference	- Some variants (K695R, homozygous M694V) are linked to a more progressive course; however, the overall impact is inconsistent	Exacerbation (not clearly predisposing)
RA	- Chronic IL-1β/TNF-α/IL-6–driven inflammation - Possible HLA–*MEFV* epistasis	M694V, M680I,V726A, and E148Q	- In numerous cohorts, *MEFV* frequency is similar in patients and controls - In a Moroccan cohort, *MEFV* mutations were more prevalent in RA patients: carrier parents of FMF patients show higher RA incidences	*- MEFV* carriers may have more severe RA symptoms, more febrile episodes; but the effect on the disease onset is uncertain	Exacerbation
SS	CD4+ T-cell–mediated glandular inflammation, elevated IL-18 in FMF, and shared systemic inflammation	M694Vand M694I	Very rare, mostly case reports; underreported	Colchicine reduced inflammatory cytokines in cases; but no clear consistent phenotype	Rare/uncertain (possibly coincidental)
CD	- Overlapping gut inflammation - IL-1/NLRP3 interface - Clinical symptoms overlap (abdominal pain and diarrhea)	E148Q and M680I	- Pediatric studies show no enrichment of *MEFV* in CD and no higher incidences of CD in FMF - Larger cohort with ESPGHAN criteria reported no CD prevalence in FMF patients	- No consistent increase in CD risk or severity among FMF - Co-occurrence may complicate diagnosis	No significant association
Psoriasis	- Cutaneous manifestations, systemic inflammation, dysregulated pro-inflammatory cytokines (IL-1, IL-17, TNF-α), Th17 cell activation, and keratinocyte stimulation	M694V	- FMF cohorts report higher psoriasis prevalence (~12–20%) - Psoriasis is more prevalent in adult FMF patients than juveniles	- FMF inflammatory milieu may promote psoriatic lesions, with clinical monitoring being advised	Increased prevalence/Exacerbation
HT	- Possible shared cytokine dysregulation - FMF-associated inflammation may trigger thyroid autoimmunity	M694V and E148Q	- Case reports describe FMF+HT coexistence - Higher thyroid autoantibodies levels in a small cohort of FMF patients - Larger pediatric and adult cohorts show no increased prevalence (1.6% HT in FMF, within general population range)	- Thyroid autoimmunity, hypothyroidism, and reported use of levothyroxine therapy	No significant association
T1DM	Elevated IL-1, IL-6, TNF-α, Th17 cell activation, NLRP3 inflammasome involvement, and systemic inflammation	E148Q, M680I, M694I, and V726A	- Small case–control: no significant *MEFV* enrichment - Co-occurrence largely rare and case-based	- FMF may exacerbate inflammatory symptoms in T1DM patients (fever, abdominal/chest pain, arthralgia) - Colchicine alleviates FMF-related symptoms	Exacerbation/rare co-occurrence
MetS	Chronic low-grade inflammation, elevated IL-1β, IL-6, TNF-α, macrophage infiltration in adipose tissue, and NF-κB activation.	R202Q	- FMF cohorts show higher MetS frequency (~43% vs ~29% controls) - Children with FMF showed increased insulin resistance even in attack-free periods	- Patients show increased insulin resistance - Hyperuricemia could serve as potential marker - Potential long-term risk for T2DM, hypertension, and dyslipidemia	Proinflammatory predisposition
Amyloidosis	- Chronic inflammation in FMF leads to elevated SAA, promoting amyloid fibril formation - Proinflammatory cytokines (IL-1β, TNF-α, IL-6) drive SAA production	M694I, M694V, M680I, E148Q, and I692Del	- FMF cohorts show higher *MEFV* mutation frequency in amyloidosis patients - Population-specific variants such as M694I in Algeria are strongly associated with renal AA amyloidosis - M694V and E148Q show variable effects depending on population	- Life-threatening AA amyloidosis and amyloid goiter - Colchicine can prevent AA amyloidosis, but individual risk cannot be precisely predicted	Directly causal
NAFLD	Chronic systemic inflammation in FMF leads to elevated proinflammatory cytokines (IL-1β, TNF-α, IL-6) which drive hepatic inflammation, insulin resistance, and NF-κB activation	M694V	One biopsy case suggests increased NAFLD/NASH incidences in FMF while other matched studies show no prevalence difference	- Some FMF patients progress to NASH/ Cirrhosis - Regular liver function monitoring and colchicine intake are recommended	Potential predisposition

IBD, Inflammatory Bowel Disease; CD, Crohn’s Disease; UC, Ulcerative Colitis; SLE, Systemic Lupus Erythematosus; CRP, C-Reactive Protein; SAP, Serum Amyloid P; MS, Multiple Sclerosis; IL-1, Interleukin-1; TNF-α, Tumor Necrosis Factor-alpha; IFN-γ, Interferon-gamma; RA, Rheumatoid Arthritis; SS, Sjögren’s Syndrome; CD, Celiac Disease; HT, Hashimoto’s Thyroiditis; T1D, Type 1 Diabetes; MetS, Metabolic Syndrome; SAA, Serum Amyloid A; AA, Amyloidosis; NAFLD, Non-Alcoholic Fatty Liver Disease; NF-κB, Nuclear Factor kappa-light-chain-enhancer of activated B cells; NASH, Non-Alcoholic Steatohepatitis.

## 4 Conclusion

FMF is not considered a typical autoinflammatory disease, as increasing evidence highlights its frequent coexistence with both autoimmune and metabolic disorders. While some studies have demonstrated strong associations, others have yielded inconsistent results, reflecting the complex interplay between *MEFV* genetic variants, epigenetic factors, and environmental influences. Nonetheless, these co-occurrences suggest shared underlying mechanisms, such as innate immune dysregulation, inflammasome activation, and chronic metabolic inflammation.

Clinically, this underscores the importance of vigilant monitoring in both directions: healthcare providers should consider the possibility of FMF in patients presenting with autoimmune or metabolic conditions, and conversely, remain alert to potential comorbidities when diagnosing FMF. Incorporating genetic testing for key *MEFV* variants (such as M694V, E148Q), especially in patients with atypical presentations or overlapping inflammatory features, can help identify high-risk individuals and guide early and tailored interventions. Furthermore, integrating metabolic and autoimmune screening into FMF management may improve long-term outcomes and prevent secondary complications.

Despite recent advances, significant gaps remain. Future research should include longitudinal cohort studies to clarify the impact of *MEFV* variants on comorbidity development, functional studies to link specific mutations to pathogenic mechanisms, and multi-omics approaches combining genetic, epigenetic, transcriptomic, and metabolomic data, to uncover shared inflammatory pathways. Additionally, efforts to identify novel biomarkers could enable earlier detection of comorbidities and refine risk stratification.

Ultimately, viewing FMF within the broader context of inflammation not only enhances our understanding of its pathogenesis but also paves the way for more precise and effective patient care.

## Glossary

AID: Autoinflammatory disease
AD: Autoimmune disease
mAID: Monogenic autoinflammatory disease
FMF: Familial Mediterranean Fever
MEFV: Mediterranean Fever gene
PYD: Pyrin domain
ASC: Apoptosis-associated speck-like protein
IL: Interleukin
bZIP: Basic leucine zipper
miRNA: MicroRNA
CD: Crohn’s disease
UC: Ulcerative colitis
IBD: Inflammatory bowel disease
IBDU: Inflammatory bowel disease unclassified
IC: Indeterminate colitis
NLRP3: NOD-like receptor family pyrin domain-containing 3
CARD15: Caspase activation and recruitment domain family member 15
NOD2: Nucleotide-binding oligomerization domain-containing protein 2
NF-κB: Nuclear factor kappa-light-chain-enhancer of activated B cells
TNF: Tumor necrosis factor
SLE: Systemic lupus erythematosus
CNS: Central nervous system
anti-dsDNA: Anti–double-stranded DNA antibody
anti-Sm: Anti-Smith antibody
anti-RNP: Anti–ribonucleoprotein antibody
anti-Ro/SSA: Anti–Sjögren’s syndrome antigen A antibody
anti-La/SSB: Anti–Sjögren’s syndrome antigen B antibody
BAFF: B-cell activating factor
SAP: Serum amyloid P component
CRP: C-reactive protein
snRNP: Small nuclear ribonucleoprotein
HLA: Human leukocyte antigen
MS: Multiple sclerosis
TNFRSF1A: Tumor necrosis factor receptor superfamily member 1A
TRAPS: TNF receptor–associated periodic fever syndrome
IFN-γ: Interferon gamma
PP: Primary progressive (multiple sclerosis subtype)
RR: Relapsing–remitting (multiple sclerosis subtype)
CCR5: C-C chemokine receptor type 5
RA: Rheumatoid Arthritis
HLA: Human Leukocyte Antigen
SS: Sjögren’s Syndrome
CD: Celiac Disease
AGA: Anti-Gliadin Antibodies
EMA IgA: Anti-Endomysium Antibodies Immunoglobulin A
tTG IgA: Tissue Transglutaminase Immunoglobulin A
ESPGHAN: European Society for Paediatric Gastroenterology Hepatology and Nutrition
Th: T helper
T1DM: Type 1 Diabetes Mellitus
ATD: Autoimmune Thyroid Disease
MetS: Metabolic Syndrome
IR: Insulin Resistance
T2DM: Type 2 Diabetes Mellitus
IRS-1: Insulin Receptor Substrate-1
AP-1: Activator Protein 1
AA: Amyloid A
SAA: Serum Amyloid A
S-AA: Secondary Amyloidosis (non-FMF related)
AA-FMF: Amyloidosis-associated FMF2
NAFLD: Nonalcoholic Fatty Liver Disease
NASH: Nonalcoholic Steatohepatitis
SOCS3: Suppressor of Cytokine Signaling
